# Transient Cognitive Impairment in Epilepsy

**DOI:** 10.3389/fnmol.2018.00458

**Published:** 2019-01-07

**Authors:** Silvia Landi, Luigi Petrucco, Federico Sicca, Gian Michele Ratto

**Affiliations:** ^1^NEST, Istituto Nanoscienze-CNR and Scuola Normale Superiore, Pisa, Italy; ^2^Graduate School of Systemic Neurosciences, Ludwig Maximilian University of Munich (LMU), Munich, Germany; ^3^Department of Developmental Neuroscience, Fondazione IRCCS Stella Maris, Pisa, Italy

**Keywords:** epileptic encephalopathy, cognitive impairment in mental disorder, interictal epileptiform discharges, EEG, animal models of epilepsy

## Abstract

Impairments of the dialog between excitation and inhibition (E/I) is commonly associated to neuropsychiatric disorders like autism, bipolar disorders and epilepsy. Moderate levels of hyperexcitability can lead to mild alterations of the EEG and are often associated with cognitive deficits even in the absence of overt seizures. Indeed, various testing paradigms have shown degraded performances in presence of acute or chronic non-ictal epileptiform activity. Evidences from both animal models and the clinics suggest that anomalous activity can cause cognitive deficits by transiently disrupting cortical processing, independently from the underlying etiology of the disease. Here, we will review our understanding of the influence of an abnormal EEG activity on brain computation in the context of the available clinical data and in genetic or pharmacological animal models.

## Introduction

Epilepsy is a heterogeneous disorder that includes a great variety of phenotypes and pathological manifestations, all characterized by enhanced neuronal excitability due to an impaired balance between excitation and inhibition (E/I). In this review article, we will deal mostly with subclinical interictal activity, that can impact on everyday life by causing transient alterations of cortical computation. First, we will attempt to delineate the difference between chronic cognitive decline and transient cognitive deficits due to brief episodes of altered network excitability in humans. These studies require to correlate the EEG recording with the outcome of the cognitive task, to verify whether the presence of interictal epileptic discharges (IEDs) causes a temporary deficit. Second, we will review the available animal models that can be used to analyze brain computation during chronic and transient hypersynchronous activity. Addressing these topics can foster a better understanding of the still ambiguous role of interictal epileptiform activity in modulating development and cognition. This knowledge is necessary to assess the utility of treating interictal EEG abnormalities to reduce cognitive impairment.

## Epilepsy and Co-morbidity With Neuropsychiatric Disorders

**Figure 1:**
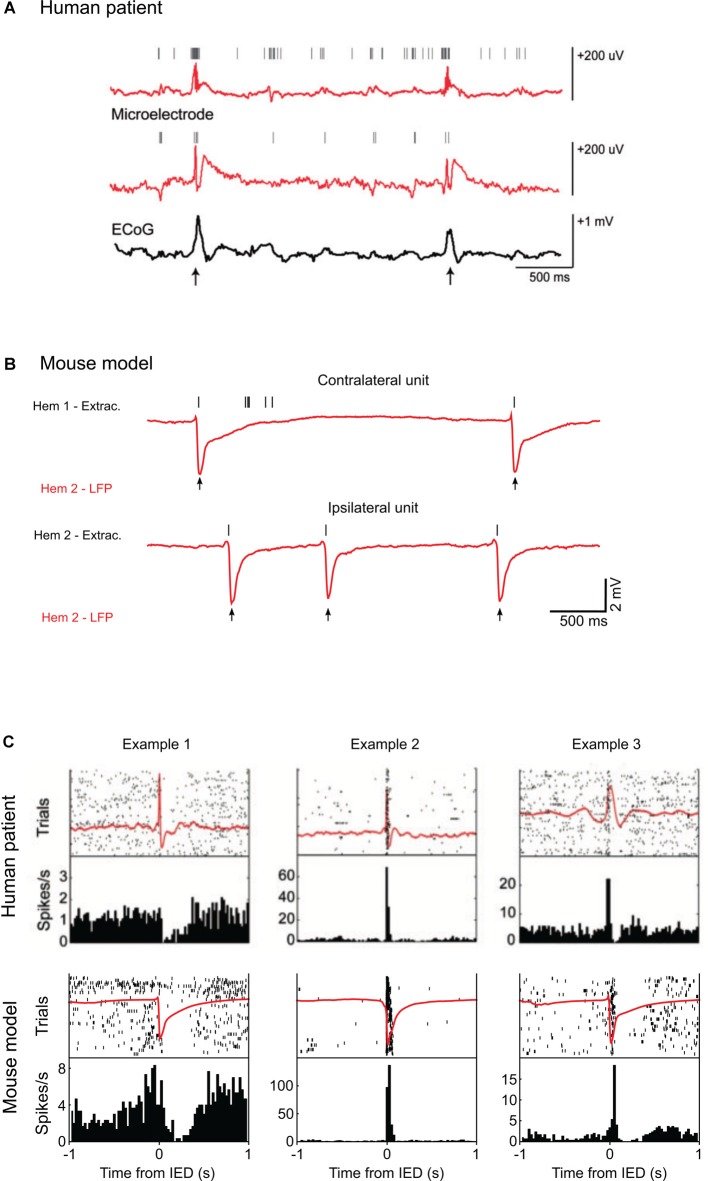
Single-unit activity during interictal epileptic discharges (IEDs) in humans and mice. **(A)** Top: example of local field potential (LFP) recordings from two microelectrodes (red) and simultaneous corticography (ECoG; black) in patients with medically intractable focal epilepsy during epilepsy monitoring. Vertical marks indicate single units recorded by the two electrodes. Action potentials from the two single units cluster around the IEDs (arrows). **(B)** Examples of LFP recordings (red) from layers 2/3 of the mouse visual cortex after unilateral superfusion of bicuculline methiodide. Vertical marks (black) indicate the spikes recorded in the loose patch configuration from the contralateral, untreated hemisphere (above) and from the cortical patch treated with bicuculline (below). In the IED focus, spikes are present only at the time of the IEDs (arrows) whereas in the opposite hemisphere other spikes appear around the events. **(C)** Top: IED-locked raster plots and peri-event time histograms for three example units from the recordings in a human patient. The average LFP is shown in red. The cells display distinct firing modulations around the hypersynchronous event. Below, the same representation is used to show average LFP and firing activity from three units recorded in the mouse during bicuculline induced IEDs. Examples 1 and 3 come from contralateral units, Example 2 from an ipsilateral unit. The firing frequency is similarly modulated by the IEDs in both in the human and mouse recordings. Human data are reproduced with permission from Keller et al. (2010). Mouse data are reproduced with permission from Petrucco et al. (2017).

Epileptic seizures are transient episodes of excessive synchronous activity in the brain (Noachtar et al., 1999; Chang and Lowenstein, 2003; Shorvon, 2011; Fisher et al., 2014; Trevelyan, 2016; Wang et al., 2017). They encompass a diverse group of events ranging from minimal clinical manifestations, e.g., brief and nearly undetectable losses of consciousness, to vigorous episodes of muscular shaking that can result in physical injuries. Epileptic events can also be transiently induced by brain trauma, injuries, drugs, temperature, hypoxia and other deviations from normal brain homeostasis (Goldberg and Coulter, 2013). Even if epilepsy is a worldwide pathology affecting more than 65 million people (Ngugi et al., 2010), we are still far from a clear understanding of the common mechanisms underlying its very diverse spectrum of presentations (Dichter, 2009; Pitkänen and Lukasiuk, 2009; Vezzani, 2014). The analysis of the EEG of patients with recurrent seizures demonstrates the existence of abnormal electrophysiological events in between seizures, defined as “subclinical” for the apparent absence of clinical correlates. These events have been named interictal epileptiform discharges (IEDs; see Figure 1; Noachtar et al., 1999; de Curtis and Avanzini, 2001; Pillai and Sperling, 2006; Fisher et al., 2014). Interictal discharges are commonly divided on the base of the electroencephalographic signature of the event into four major categories: sharp waves, spikes, sharp waves/spikes-and-slow-waves and multiple spikes-and-slow-waves (Kooi, 1966; Gotman, 1980; Kane et al., 2017). As the name indicates, IEDs are abnormal events temporally distinct from ictal events, and are not directly implicated in the genesis of ictal events (Noachtar et al., 1999; Avoli et al., 2006; Fisher et al., 2014).

IEDs are caused by the simultaneous firing of a large number of cortical neurons that produces a paroxysmal depolarization shift in neuronal cohorts (Matsumoto and Marsan, 1964; Prince, 1968; Schwartzkroin and Wyler, 1980). Since seizures are infrequent in the majority of patients, their detection by EEG is painstaking and may require prolonged recording sessions; for this reason, the detection of IEDs is often the first-line diagnostic tool. The detailed description of interictal activity provides the clinicians with insights into the nature of the associated epileptic pathology. Moreover, the different patterns and localization of interictal events are often predictive for the localization of the epileptogenic focus (Berg and Shinnar, 1991; Pillai and Sperling, 2006).

A groundbreaking study (Bridgers, 1987) determined that in a large cohort of psychiatric patients affected by depression, mania, personality disorders, suicidality without depression, nonpsychotic explosive behavior, the probability of epileptiform activity was higher than in the general population and many studies then revealed EEG anomalies in these type of patients (Cook et al., 1986; Weilburg et al., 1995; Hughes, 1996; Hayashi et al., 2010; Badrakalimuthu et al., 2011; Beletsky and Mirsattari, 2012; Gao and Penzes, 2015; Lee et al., 2017). Moreover, it was found that IEDs were present in up to 60% of school-aged children affected with hyperactivity disorders (Richer et al., 2002; Holtmann et al., 2003; Silvestri et al., 2007), while EEG alterations occurred in only 2%–3% to 6.5% of healthy children (Barkmeier and Loeb, 2009; Borusiak et al., 2010). Similarly, some studies report a high rate of EEG abnormalities, ranging from 7% to 70%, in individuals with autism spectrum disorders (ASDs), even without ictal manifestations (Spence and Schneider, 2009; Valvo et al., 2016). A natural conclusion that can be attained by these observations is that anomalies of the neural circuitry driving diverse brain dysfunctions may share some of the pathogenic mechanisms of epilepsy.

## Molecular and Physiological Mechanisms of Pathological Hyperexcitability in Epilepsy and Autism

The correct regulation of inhibition is a necessary condition for normal brain processing (Van Vreeswijk and Sompolinsky, 1996; Hensch and Fagiolini, 2005; Mariño et al., 2005; Trevelyan and Watkinson, 2005; Buzsáki et al., 2007; Atallah and Scanziani, 2009; Baroncelli et al., 2011; Vogels et al., 2011; Yizhar et al., 2011; Haider et al., 2013; Nelson and Valakh, 2015; Dehghani et al., 2016; Denève et al., 2017). In the healthy brain, the recruitment of inhibitory interneurons by feedforward and feedback excitatory connections ensures that local inhibition closely follows excitation in a given cortical area (Okun and Lampl, 2008; Isaacson and Scanziani, 2011). Neuronal networks are constantly challenged by alterations of E/I and the brain employs several homeostatic mechanisms to adjust net excitability in order to maintain network activity within the physiological range and to prevent saturation (see Turrigiano, 2011). The slightest deviation from this condition can lead to dramatic outcomes: a reduction of excitation may drive the induction of a comatose state, while reduction of the inhibitory feedback can result in network hyperexcitability and epileptiform activity (Dudek and Sutula, 2007; Trevelyan et al., 2013). Even if inhibitory cells are only 25% of all neurons, their role is essential in modulating network activity (for review Schmidt-Wilcke et al., 2017). When inhibition is reduced, and the cortex becomes hyperexcitable (Dichter and Ayala, 1987), neurons may display a wide range of abnormal behaviors. These anomalies can go from a mildly diminished selectivity in the response to different stimulus features (Sillito, 1975), to massive, hypersynchronous events that entrain the population and functionally disconnect it from its afferent inputs. The interplay between E/I is essential to ensure proper gain control and normalization of neuronal activity and to refine the timing of principal cell firing (Chance et al., 2002; Isaacson and Scanziani, 2011). Obviously, in the absence of counteracting inhibition, threshold depolarization would be reached with a much weaker excitatory input, increasing the error and variability of the response. It is important to note that the maintenance of the E/I balance is not a static, hardwired mechanism but a deeply dynamic process maintained by constant plastic adjustments (Froemke et al., 2007; Dorrn et al., 2010).

Given this background, it is not surprising that a defective feedback between E/I has been recognized as one of the key factors in the insurgence of many central nervous system (CNS) pathological conditions (Fernandez and Garner, 2007; Gao and Penzes, 2015; Lee et al., 2017). Indeed, it is known that inhibitory GABAergic signaling is hampered in models of ASD (Gibson et al., 2008; Gogolla et al., 2009; Chao et al., 2010; LeBlanc and Fagiolini, 2011; Yizhar et al., 2011; Bateup et al., 2013; Selimbeyoglu et al., 2017), which justify the co-morbidity between ASD and various forms of hyperexcitability. Since the disruption of GABA signaling increases network gain, this contributes to amplify background noise thus interfering with neuronal coding. Not surprisingly, the potentiation of GABA signaling is a prime pharmacological target that can result in a rescue of the phenotype (Lewine et al., 1999; Tuchman and Rapin, 2002; Levisohn, 2007; Gatto and Broadie, 2010; Bolton et al., 2011; Pizzarelli and Cherubini, 2011; Gilby and O’Brien, 2013; Tuchman, 2013; Cellot and Cherubini, 2014; Jeste and Tuchman, 2015; Buckley and Holmes, 2016). In contrast, other studies suggest that blocking the response to GABA can ameliorate cognitive impairment such as in Down syndrome (Kleschevnikov et al., 2004; Fernandez et al., 2007; Belichenko et al., 2009), in Rett syndrome (Dani et al., 2005; Dani and Nelson, 2009), in Angelman syndrome (Mabb et al., 2011) and in phenylketonuria (De Jaco et al., 2017). These opposing scenarios could be partly explained by the fact that GABA response polarity is not univocal, but it is determined by intracellular Cl^−^ concentration, which in turn is finely regulated by the interplay of leak channels and specific co-transporters (NKCC1 and KCC2; Kaila et al., 2014; Miles et al., 2012; Viitanen et al., 2010; Löscher et al., 2013). It is important to remember that at late embryonic/early postnatal stages intracellular Cl^−^ is higher than 20 mM (Sulis Sato et al., 2017) and GABA causes exit of chloride from target cells leading to membrane depolarization (Ben-Ari et al., 2012). If in these pathologies the pathways underlying chloride homeostasis do not mature properly, GABA could contribute to hyperexcitablity and GABAergic agonists would exhert a paradoxycal effect. Indeed, in Down syndrome it has been demonstrated that the maturation of Cl^−^ regulation is disrupted, thus leading to depolarizing GABA in an adult mouse model for this disorder, and the pharmacological rescue of Cl^−^ homeostasis brought about a recovery of synaptic plasticity and memory (Deidda et al., 2015). It is tempting to speculate that this mechanisms could be shared with other forms of neurodevelopmental disorders, including ASD (Cellot and Cherubini, 2014).

Crucially, pathways involved in regulating synaptic homeostasis can also be affected by the pathology itself; for example, synaptic scaling can depress N-methyl-D-aspartate (NMDA) or AMPA receptors as a consequence of the enhancement of synaptic strength (Doyle et al., 2010; Goold and Nicoll, 2010), but the homeostatic pathways converging on these receptors can be altered by pathology. For example, at least some of the complex single gene disorders associated to ASD (Rett Syndrome, Fragile X Syndrome, Tuberous Sclerosis, Aristaless Related Homeobox associated Syndromes) results in blockade of synaptic scaling, because of the disruption of transcription and/or protein synthesis (Fernandes and Carvalho, 2016). Indeed, in Fragile X the mutation of FMRP, an RNA-binding protein that regulates dendritic protein synthesis, causes the loss of a regulator of the synthesis of AMPARs in dendrites and of their insertion at the postsynaptic site, essential for the increase in synaptic strength induced by retinoic acid or by blockade of neural activity (Soden and Chen, 2010).

## Relationship Between Epileptiform Activity and Cognitive Impairment: A General Perspective

Epileptiform activity is often associated to memory impairments, mental slowing, communicative and behavioral disturbances and attentional deficits both in children and adults with epilepsy (Aldenkamp et al., 1990, 2004; Dodson and Bourgeois, 1994). An important issue for the diagnosis and treatment of epilepsy is the existence of many forms of epileptiform activity that can be difficult to classify by their EEG signature. It is also challenging to correlate subtle EEG abnormalities with the behavioral readouts of specific tasks (Blume, 2001). Indeed, patients with short non-convulsive seizures are often characterized by evanescent symptoms or by frequent transitions from IEDs to seizures. In these scenarios, the understanding of the contributions of diverse phases of epileptiform activity to cognitive impairment is especially arduous. On the other hand, a spectrum of epileptic syndromes including continuous spike wave in slow-wave sleep (CSWS) or Landau-Kleffner syndrome (LKS) clearly shows how the strong activation of IEDs during sleep may hamper cognitive functions or language, respectively (Tassinari and Rubboli, 2006). It is a clinical priority to apply proper diagnostic techniques to classify the different epileptic events, and to follow the evolution of the pathology by means of longitudinal and prospective studies (Scheltens-De Boer, 2009).

**Figure 2:**
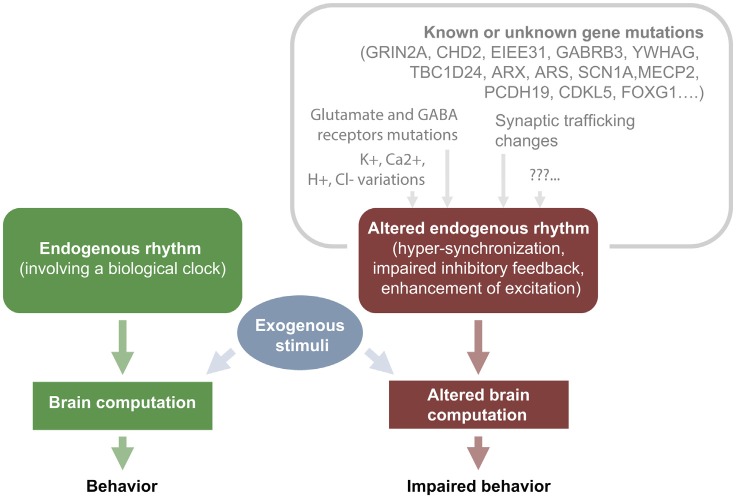
IEDs cause transient cognitive impairment: relationship between upstream mutations and hyperexcitability.

In general, it is recognized that epileptiform activities can contribute to transient or permanent deficits according to many factors, e.g., their recurrence and severity, the age of the subject and the type of therapy used to prevent seizures and its efficacy. Of course, seizures and epileptiform activity have an higher impact in subjects during development rather than in a mature brain, but is hard to establish on the basis of the available evidence whether repeated interictal spikes are more detrimental than isolated or sporadic seizures in promoting aberrant connectivity (Holmes, 2014). Long-term consequences of interictal discharges accumulating over time may produce deep effects at cognitive level impacting especially on education and learning (IQ scores over time; (Siebelink et al., 1988; Brinciotti et al., 1989; Tuchman and Rapin, 1997) possibly causing life-long effects on developing children. Several mutations can lead to hyperexcitability by enrolling different mechanistic pathways (alterations of synaptic function, alterations in connectivity, impaired metabolism and homeostasis, etc…). Then, regardless of the original causal mechanisms, hyperexcitability leads to defective neuronal computation and impaired cognition (Figure 2; for review McTague et al., 2016; Staley, 2015).

## Molecular and Physiological Mechanisms Associated to Epileptic Encephalopathies as Models of Chronic Impairment

Epileptic encephalopathies (EE) are the most representative example of how long-lasting epileptiform activities may influence the neurodevelopmental outcome. If not properly treated, EE can cause permanent deficits due to the deleterious impact of epileptiform interictal activity and seizures on the development and organization of the immature brain (Nabbout and Dulac, 2003; Hirsch et al., 2006; Holmes and Lenck-Santini, 2006). The relationship between clinical and EEG features and the degree of cognitive deterioration remains elusive and may vary greatly between different syndromes. Thus, it is often arduous to translate into therapeutic decisions the insights arising from clinical and EEG evaluations and from our understanding of the underlying pathomechanisms.

CSWS is a paradigmatic model to understand how continuous and diffuse paroxysms, lasting months or years, may impair the neuropsychological outcome. CSWS is a disorder that appears during childhood, characterized by the presence of continuous generalized spike-wave complexes during at least 85% of slow-wave sleep. Cognitive deficits associated with impairment of neuropsychological functions, reduced IQ, reduction of language, memory and motor impairment occurring in the course of this syndrome are generally assumed to be caused by repetitive interictal discharges (Praline et al., 2003). The disruption of physiologic sleep and the interference with the slow-wave activity homeostasis, with consequent impairment of neural processes and synaptic plasticity at the site of the epileptic focus, are possible pathomechanisms underlying the neuropsychological impairment characterizing the syndrome (Tassinari and Rubboli, 2006; Tononi and Cirelli, 2006). During puberty, the electrical status epilepticus in sleep (ESES) pattern progressively vanishes and the neuropsychological assessments generally improve, although many of the acquired deficits often remain. The localization of the interictal focus seems to have a role in influencing the pattern of neuropsychological derangement. For example, linguistic impairment can be observed when abnormalities are mostly located on the temporal regions, like in the acquired auditory agnosia seen in the LKS (Galanopoulou et al., 2000), or an autistic behavior can appear in relation to frontal epileptogenic foci (Deonna and Roulet-Perez, 2010). Several studies on patients treated for CSWS (De Negri et al., 1995; Yan Liu and Wong, 2000) have shown an improvement in cognitive functions in those successfully responding to treatments and showing a significant reduction of the IEDs. This suggests that the pathological activity underlying the EEG alterations, curtailed by the pharmacological treatment, is the primary cause for the onset of the cognitive symptoms. Evidences deriving from *in vitro*, *in vivo* and computational models suggest that the change from physiological to pathological oscillation seen in ESES is related to the blocking of GABA_A_-mediated inhibition between reticular thalamic neurons and thalamic neurons, which is associated with a differential activation of GABA_B_-mediated inhibition and in consequent epileptiform activity (Smith and Fisher, 1996; Beenhakker and Huguenard, 2009). Interestingly, in rat models of atypical absence epilepsy, GABA_B_-receptor antagonists have been shown to rescue the associated learning impairment (Chan et al., 2006).

Another example of EE characterized by strict correlation between the severity of EEG alterations and cognitive impairment is West Syndrome. Affected children typically have clusters of epileptic spasms within the first year of life, and show a specific interictal EEG pattern (called hypsarrhythmia) of highly disorganized background activity and multifocal slow-waves and paroxysms. Both epileptic spasms and hypsarrhythmia are typically associated with developmental regression, usually beginning with impairment of visual functions and possibly linked to hemodynamic and metabolic interferences of the IEDs on cerebral homeostasis (Siniatchkin et al., 2007). An early and appropriate treatment may, in some cases, ameliorate seizures and EEG and have a profound impact on developmental progresses (McTague and Cross, 2013).

Other forms of EE, on the contrary, show poor or no correlation between clinical or EEG severity and neurobehavioral outcome. Dravet syndrome (DS) is one of the main examples of severe EE, where the cognitive outcome does not clearly reflect seizure or IED severity. DS is primarily caused (80% of cases) by heterozygous loss-of-function mutations in the *SCN1A* gene that encodes the brain voltage-gated sodium channel type-1 (NaV1.1; Dravet et al., 2011; Marini et al., 2011). As the exons of *SCN1A* only comprise 6 kb of the gene, which is over 100 kb in size, it is conceivable that the remaining patients that have normal transcript, may be affected by mutations of the non-coding portion of the gene leading to correct expression and trafficking of NaV1.1 (Catterall, 2018). Besides febrile and afebrile pharmacoresistant seizures beginning in the first year of life, and frequent episodes of status epilepticus, affected children may develop a progressive slowing of basal EEG activities and interictal asymmetrical spikes or polyspikes and waves (Dravet et al., 2011). In addition, children with DS display a progressively worsening psychomotor delay that is only partly related to severity of epilepsy or EEG features. This suggests that the cognitive defect in DS is not only a consequence of epilepsy or EEG abnormalities themselves but may in part be due to the underlying genetic pathology, i.e., a direct role of the sodium channel dysfunction (Nabbout et al., 2013). Two additional examples of epilepsy where cognitive impairment and developmental consequences are not clearly correlated with the severity and characteristics of seizures are provided by the diseases associated to the mutations of *PCDH19* and *STXBP1*. Mutations of the *PCDH19* gene in female patients causes early onset epilepsy, in part resembling DS (Dibbens et al., 2008; Specchio et al., 2011), and often lead to cognitive phenotypes ranging from borderline to severe intellectual disability (Depienne et al., 2009; Hynes et al., 2010; Marini et al., 2010). A similar scenario is found in patients affected by *STXBP1* mutations that result in early-onset epilepsy, cognitive and motor disability and ASD (Saitsu et al., 2008; Stamberger et al., 2016).

Dysfunctions of GABA_A_ receptors have been also postulated to play important roles in EE etiology. In fact, mutations or genetic variations of the genes encoding the α1, α6, β1, β2, β3, γ2, or δ subunits (GABRA1, GABRA6, GABRB1, GABRB2, GABRB3, GABRG2 and GABRD, respectively) have been associated with early-onset epilepsies with or without febrile seizures. Evidences show that the compromised hyperpolarization mediated by altered GABA_A_ receptors is not simply caused by receptor gating abnormalities, but by complex mechanisms, including endoplasmic reticulum (ER)-associated degradation, nonsense-mediated mRNA decay, intracellular trafficking defects and ER stress (Hirose, 2014; Hernandez et al., 2016; Neske, 2016; Møller et al., 2017; Shen et al., 2017). In addition, a number of antiepileptic drugs have agonistic effects on GABA_A_ receptors (Hirose, 2014), confirming their likely contribution in the pathomechanisms of epilepsy, and their potential role as candidate targets for new therapies.

## Transient Cognitive Impairment

Early studies based on EEG recordings suggested that interictal events can also lead to transitory cognitive effects in humans (Hutt et al., 1977; Aarts et al., 1984; Shewmon and Erwin, 1988a, b). These observations have been confirmed in subsequent studies employing improved video EEG recordings synchronized with behavioral assessment (Binnie et al., 1991; Krauss et al., 1997; Liu et al., 2016; Ung et al., 2017). The general idea is that IEDs transiently disrupt the functioning of the area directly involved in the generation of the epileptiform activity, as well as connected regions (Hutt et al., 1977; Aarts et al., 1984; Shewmon and Erwin, 1988a, b; Ung et al., 2017). In general, transient cognitive impairment resulting from single discharge patterns is obviously related to the location and propagation pattern of the epileptiform event. Therefore, the identification of the cognitive phenotype requires proper behavioral assessment in terms of the specific task, its duration, and the modalities of administration.

An early study that addressed the relationship between IEDs and transient cognitive deficits was performed by analyzing short-term verbal and non-verbal memory in a cohort of subjects displaying focal/asymmetrical or symmetrical generalized epileptiform activity. Half of the patients showed transient cognitive impairment associated to the presence of IEDs. IEDs cause region-specific impairments; indeed, IEDs starting in the left hemisphere caused errors in verbal tasks, while those generated in the right one produced impairments in non-verbal tests (Aarts et al., 1984; Aldenkamp et al., 2004).

Following studies demonstrated that bursts of spike-wave patterns are usually followed by a slowing of reaction times lasting several seconds, and by total amnesia for events occurred during the EEG alteration (Porter et al., 1973; Holmes et al., 1987; Krauss et al., 1997). Importantly, this transient but conspicuous deficits can be caused even by a single focal interictal event (Aarts et al., 1984; Binnie et al., 1987; Shewmon and Erwin, 1988b; Siebelink et al., 1988; Binnie, 2003; Kasteleijn-Nolst Trenité and Vermeiren, 2005; see Petrucco et al., 2017) for an animal model correlate. The dependency of cognitive impairment on the IEDs location has been shown recently in patients with seizure onset zones lateralized on the left hemisphere, since spikes that were generated outside the seizure onset area disrupted memory encoding, while those recorded inside the critical activity focus were not detrimental for cognition (Ung et al., 2017).

**Figure 3:**
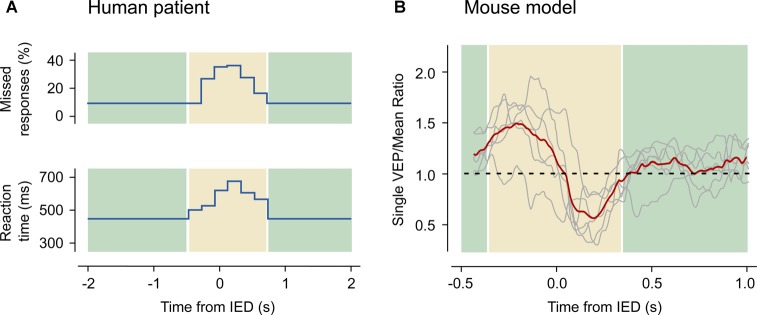
Transient cognitive impairment in humans and in mice. **(A)** IEDs interfere with a visual reaction test. Data have been acquired from a 10-year old boy suffering from partial complex seizures, tested during the occurrence of focal interictal discharges. The upper panel shows the rate of missed responses as a function of the distance between the stimulus and the nearest IED. The lower panel shows the latency of the response. Both the rate of missed stimuli and the reaction times increase in a small window around the IED event. Redrawn with data from Shewmon and Erwin (1988a). **(B)** Amplitude of visual evoked potentials (VEP) recorded in the anesthetized mouse as a function of the distance between the stimulus and the closest IED occurring in the contralateral hemisphere. A bi-directional alteration of the VEP amplitude (highlighted in the yellow area) appears when the stimulus occurs nearby an IED: if the spike precedes the stimulus the response is enhanced, while the contrary happens if the spike follows the stimulus. Reproduced with permission from Petrucco et al. (2017).

Many studies have observed that transient cognitive deficits critically depend on the timing between stimulus test and the appearance of the IEDs (Figure 3). Indeed, a single interictal discharge can affect the perception of a visual stimulus, e.g., the presentation of a stimulus at the time of an interictal event in visual cortex resulted in missed or delayed perception (Shewmon and Erwin, 1988b). A detailed characterization of the timing of the sensory deficit (Shewmon and Erwin, 1989) showed that the effect of the spikes started immediately before the deflection in the EEG trace and terminated at the end of the slow-wave. This finding led the authors to the conclusion that the long lasting slow-wave, and not only the paroxysmal spike, can affect the perception of the stimulus (see Figure 3A). The critical correlation of the IEDs timing and the transient cognitive deficit is also present for memory tasks. An important study was performed on patients undergoing the procedure for preoperative seizure localization that were implanted with deep electrodes and were tested for memory maintenance and retrieval during the electrophysiological recording (Kleen et al., 2013). These data showed a decrease of the likelihood of correct responses when the IEDs occurred during the memory retrieval period, thus suggesting a direct link between the pathological activity and cognitive impairment.

The dialogs between IEDs and cognitive functions are somehow bidirectional, since it has long been recognized that performing a cognitive task can affect the frequency of interictal paroxysms. For example, an old study (Schwab et al., 1941) determined that generalized spike and wave discharges could be reduced by light or sound stimuli delivered during a reaction time task. Recently, it has been shown that during a test in which images already archived in memory are recognized, there is a significant reduction in the IEDs rate in the amygdala, hippocampus and temporal cortex. Interestingly, during the visual task all these structures were interested by a loss of power in the theta, alpha and beta bands, as well as by an enhanced power in the gamma band. This suggests that the reorganization of network synchronization during the task may correlate with a negative modulation of local IEDs frequency (Matsumoto et al., 2013).

Finally, the detection of temporary cognitive impairment depends on the type and duration of the test (Mirsky and Van Buren, 1965; Aarts et al., 1984; Aldenkamp et al., 2004). Indeed, the beginning of a cognitive task is usually associated to an activating “arousal” effect which can suppress IEDs discharges, causing a global improvement of the test performance (Aarts et al., 1984; Binnie et al., 1987; Stores, 1990). Thus, simple motor tasks and other tests that do not require the processing of complex sets of information are less affected in their execution than more cognitively demanding tests.

## Animal Models of Chronic and Transient Cognitive Impairment

Animal models are essential to understand the molecular and physiological mechanism linking the transient electrophysiological disturbance to the alteration of cortical processing. However, the literature on animal models is relatively poor in comparison with the wealth of data provided by the clinics, possibly because of the difficulty of generating standardized models of interictal epileptiform activity (Grone and Baraban, 2015; see Table 1).

**Table 1:** Cognitive deficits in models of interictal epileptic discharges (IEDs).

Models	Type of treatment	Cognitive effect	Putative molecular mechanisms	References
**Chronic models**				
Awake macaque	Local intracortical administration of bicuculline in the primary visual cortex	Larger amplitude of visual evoked potentials and enhancement of single-cell activity	Competitive antagonist of GABA_A_ receptors	Schroeder et al. (1990)
Rat prefrontal cortex (PFC) in adult animals	Five days-injections starting from P21 with bicuculline	Increment in short-term plasticity in the PFC. No working memory deficits, but marked inattentiveness.	Competitive antagonist of GABA_A_ receptors	Hernan et al. (2014a); Hernan et al. (2014b)
Freely moving rats	Lithium i.p. followed by subcutaneous pilocarpine injection	Reduced stability of place cells. Impaired spatial memory	Non-selective agonist of muscarinic receptor	Liu et al. (2003)
Sprague-Dawley rats	Pilocarpine injection	Impaired performances in memory retrieval using a hippocampal-dependent operant behavior task	Non-selective agonist of muscarinic receptor	Kleen et al. (2011)
Rat pups	Inhalation of fluorothyl (4 h every day for 10 days)	Deficits in spatial memory (Morris water maze; four-trial radial arm water maze) and in LTP	Blockade of GABA_A_ receptors, with the addition of opening of sodium channels and activation of the cholinergic system	Khan et al. (2010)
Rat model of temporal lobe epilepsy	Kindling or repeated single electrical pulses to the hippocampal commissure	Impaired memory consolidation at the hippocampus	N-methyl-D-aspartate receptor-activated changes in gene expression	Gelinas et al. (2016)
Mouse visual cortex	Tetanus neurotoxin (TeNT)-induced focal epilepsy	Reduction of reliability of visual responses; impairment of visual acuity	Blockade in the release of GABA and glycine	Vannini et al. (2016)
**Transient models**				
Cat auditory cortex	Focal application of penicillin	Altered processing of sensory evoked potentials	Inhibition of GABA release/action	Schraeder and Celesia (1977)
Ferret visual cortex	Iontophoretic injection of bicuculline	IEDs triggered by specific visual stimulation pattern according the epileptic focus location	Competitive antagonist of GABA_A_ receptors	Schwartz and Bonhoeffer (2001) and Schwartz (2003)
Mouse visual cortex	Local cortical superfusion of bicuculline	Silencing of the contralateral cortex during IEDs. Disturbances of sleep-slow wave activity and impaired vision.	Competitive antagonist of GABA_A_ receptors	Petrucco et al. (2017)

A large body of studies on slices have employed different pharmacological strategies to induce interictal activity (Fisher, 1989; Pitkänen and McIntosh, 2006; Barkmeier and Loeb, 2009; Avoli and Jefferys, 2016): GABA_A_ receptor blockade (Meldrum and Horton, 1971; Collins and Caston, 1979; de Curtis et al., 1998; Uva et al., 2005), low extracellular magnesium concentration (Tancredi et al., 1990), low extracellular calcium (Lian et al., 2001), kainic acid (Medvedev et al., 2000) and 4-aminopyridine (Lévesque et al., 2013). *In slice* studies have provided valuable information on the cellular mechanisms at the basis of IED generation and propagation, but they cannot fully recapitulate what happens in the animal brain *in vivo* and cannot provide a causal link between the ectopic activity and the disruption of cortical computation.

In intact animals, focal IEDs have been generated by electrical stimulation (Collins and Caston, 1979; Gelinas et al., 2016) or after systemic or focal administration of inhibitors of GABA activity (Meldrum and Horton, 1971; Schroeder et al., 1990; Schwartz and Bonhoeffer, 2001; Hirase et al., 2004; Ma et al., 2004; Hernan et al., 2014a, b), kainic acid (Berdyyeva et al., 2016) and pilocarpine (Kleen et al., 2011). Finally, a focal model of epileptic activity has been obtained by the localized microinjection of tetanus toxin (TeNT; Brener et al., 1991; Nilsen et al., 2005; Vannini et al., 2016) that causes an increase of the E/I ratio because of a strong blockade of inhibitory synapses (Ferecskó et al., 2014).

When discussing animal studies, one should make an important distinction between chronic and transient models of IED activity. The chronic models are defined by prolonged treatments that may lead to permanent EEG irregularities. In these models, the assessment of cognitive functions generally occurs days or weeks after treatment and probes the effects of the IED activity, if still present, and of the homeostatic response of the brain to the treatment. However, in chronic models, it is difficult to understand whether the observed cognitive deficits are directly due to anomalous activity or to cortical rearrangements that have occurred during the response to the induction that cause steady state alterations of cortical computation. In contrast, transient models take advantage of an acute manipulation that leads immediately to IEDs, and allow assessing their short-term effects on cognitive functions before the onset of any long-term homeostatic change.

### Chronic Models

A useful example of chronic model is provided by rat pups exposed for 10 days to a low dose of flurothyl (an inhalant chemoconvulsant) for 4 h (Khan et al., 2010). Pups developed IED activity without seizures during the treatment and activity returned to normality after inhalation ending. When the rats reached adulthood, behavioral and electrophysiological testing revealed impairments of spatial memory and long-term potentiation. Anatomical assessment of the hippocampus showed a reduction of newly born cells without an increase in apoptosis at a single time point; however, it was not clear in this study if neurogenesis or apoptosis affected excitatory or inhibitory neurons, rather than glia. In a previous study on the same model by the same group, it was reported the loss of inhibitory GABAergic interneurons, while epileptiform activity did not influence glutamatergic synapse maturation, thus supporting the hypothesis of activity independence of the development of AMPA/NMDA signaling (Isaeva et al., 2006). These studies suggest that the transient IEDs evoked by the treatment have led to long term changes in the hippocampal structure and to cognitive impairment.

Although flurothyl inhalation can be precisely regulated to provide varied degrees of ictal or interictal activity (Modica et al., 1990), its long-term effects are very complex involving a number of factors, including changes in the brain content of DNA (Wasterlain, 1976), increased expression of cyclooxygenase-2 (Kim and Jang, 2006), changes in the intrinsic excitability and postsynaptic composition (Villeneuve et al., 2000; Swann et al., 2006) and impairment of dendrite development (Nishimura et al., 2011). Thus, the interpretation of the flurothyl model is not straightforward, thus preventing a clear causal relationship between IEDs in early development and cognitive impairment in adulthood.

Other pharmacological models that affect directly the E/I balance may provide a more direct causal link between abnormal activity and cognitive deficit. The obvious target for this manipulation is the GABA_A_ receptor, which can be modulated by a rich pharmacological toolbox. Bicuculline methiodide is a competitive agonist that, upon local delivery, causes the onset of IEDs resembling the activity appearing in lesional human epilepsy (Noachtar et al., 1999) and in idiopathic benign partial epilepsies of childhood (de Curtis et al., 1998). A recent study generated a rat model of focal IEDs caused by repeated injections of bicuculline into the prefrontal cortex (PFC) starting from P21 (Hernan et al., 2014a). Upon reaching adulthood, the treated rats showed an increment of short-term plasticity in the PFC, deficits in social behavior and marked absence of attention without significant increment of anxiety or of hyperactivity. This study concluded that the focal IED activity, caused by GABAergic blockade during early-life, disrupted the circuitry of the PFC thus leading to long-term effects on behavior after the ending of IEDs. However, the co-administration of adrenocorticotropic hormone, a drug widely used to treat seizures starting early in life (Rosati et al., 2017), produced a modest amelioration of the attention deficit in adulthood even if it did not reduce IEDs. This result suggested a partial uncoupling between the acute electrophysiological response and the long-term cognitive deficits.

The long-term effects of epileptiform activity have been recently studied in the visual cortex after focal treatment with TeNT. In this case, frequent spike bursts were followed by an upregulation of GABA markers, possibly suggesting a compensatory response. Sensory processing is a paradigmatic example of cortical computation that relies on a finely tuned negative feedback operated by inhibitory interneurons to normalize the wide range of external stimuli to the limited dynamic range of cortical coding. Interestingly, this treatment caused a dendritic rearrangement different from that one observed after flurothyl inhalation, since length complexity of dendrites increased. Visual responses were less reliable in comparison to controls, possibly because of the degradation of the signal to noise ratio of the network, as also suggested by increased firing rate in resting state (Vannini et al., 2016).

Finally, in a kindling model of temporal lobe epilepsy, the communication between hippocampus and medial PFC (mPFC) was altered by IEDs, determining the disruption of the spatial navigation memory in freely behaving rats; moreover, there was a correlation between the degree of memory impairment and the frequency of hippocampal IEDs capable to elicit spindles in the mPFC (Gelinas et al., 2016).

### Transient Models

Transient models of activity provide the opportunity to study in a normal brain the direct effect of IEDs on cognitive functions. In these models, there are no long-term effects caused by previous treatments, and the anomalies can be ascribed only to neurons being hijacked by the hypersynchronous firing occurring at each IED. In the past, a large number of investigators has been using acute superfusion with convulsants, such as penicillin or bicuculline, to address *in vivo* the physiological mechanisms of IEDs (Goldenshohn and Purpura, 1963; Matsumoto and Marsan, 1964; Prince, 1968; Collins and Caston, 1979). These early studies provided us with the first detailed description of the anatomy of an IED (reviewed in Dichter and Ayala, 1987).

Acute pharmacological models are ideal to study the interaction between focal IEDs and its effects on cortical computation, since the timing and intensity of the IED activity can be carefully regulated while probing cortical computation. Indeed, similar manipulations have been used to address how the abnormal recruitment affected physiological sensory processing of both auditory (Schraeder and Celesia, 1977) or visual stimuli (Ebersole and Levine, 1975; Schwartz and Bonhoeffer, 2001; Schwartz, 2003).

A recent study from our laboratory (Petrucco et al., 2017) addressed how cortical processing can be transiently disrupted even in areas far from the interictal focus. IEDs were triggered in the visual cortex of the anesthetized mouse by strictly localized superfusion of bicuculline (Figure 1). As expected, in the treated territory computation was abolished, since all pyramidal neurons were recruited by IEDs. This effect extended to the contralateral cortex, because the IED initially facilitated firing in this area but, after about 100 ms, completely silenced the contralateral cortex for almost 300 ms. Moreover, visual evoked responses were affected depending on the relative timing between stimulus presentation and contralateral spike burst (Figure 3). Therefore, the timing of the hypersynchronous spike defines a temporal window of impaired cognition in the cortical territory recruited by the event. This was accompanied by a secondary deficit caused by the propagation of the hypersynchronous activity to connected brain areas. These rodent data correlates with findings from coupled EEG and fMRI recordings in human subjects showing changes of the BOLD signal in remote structures far from the epileptic generator (Kobayashi et al., 2005, 2006). Indeed, several of the clinical evidences discussed above indicate that, while the location of focal IEDs determines the nature of the deficit, the connected areas are also affected by the propagated spikes and contribute to the overall cognitive deficit (Ung et al., 2017).

Together, these studies prove that IEDs disrupt endogenous rhythms and affect brain information processing even in absence of the circuitry rearrangements proper of chronic epilepsy and independently from epileptic foci.

## Conclusions

It is fair to conclude that several clinical evidence and the acute animal models suggest that IED activity interfere with brain computation in the focus and in connected areas, contributing to the overall cognitive impairment. Therefore, it is important to understand whether IEDs should receive pharmacological treatment even in absence of seizures. The answer is not obvious, since antiepileptic drugs can be associated with cognitive side effects (Ben-Menachem et al., 1989; Aldenkamp et al., 2004; Eddy et al., 2012; Perucca and Gilliam, 2012; Glauser et al., 2013) and a careful cost/benefit analysis is required for each specific case. However, curing IEDs is possible and sometimes it results in cognitive improvement, especially ameliorating educational development in children (Besag, 1995; Noachtar et al., 1999; Pressler et al., 2005; Beghi et al., 2013; Kleen et al., 2013). This is true not only for EE like CSWS, where IEDs persist over months or years thus chronically impairing the physiological patterns of EEG sleep activities, but also for epilepsies where the IEDs are less evident, especially some focal epilepsies (Aldenkamp et al., 2004; Sánchez Fernández et al., 2015). The demonstration that the effects of IEDs on neuronal processing are restricted to a brief temporal window around each event, underlines the importance of matching the behavioral readout of transient cognitive impairments to the ongoing EEG identification of IEDs. A better understanding and evaluation of the impact of IEDs on cognition will allow defining the most appropriate pharmacological strategies to treat not only seizures, but also those interictal events that may affect intellectual development and functions.

## Author Contributions

All authors contributed to the discussion and preparation of the manuscript.

## Conflict of Interest Statement

The authors declare that the research was conducted in the absence of any commercial or financial relationships that could be construed as a potential conflict of interest.
